# A theoretical foundation for relating the velocity time integrals of the left ventricular outflow tract and common carotid artery

**DOI:** 10.1007/s10877-022-00969-0

**Published:** 2023-01-10

**Authors:** Jon-Emile S. Kenny

**Affiliations:** 1 Health Sciences North Research Institute, 56 Walford Rd, Sudbury, ON P3E 2H2 Canada; 2 Flosonics Medical, Toronto, ON Canada

*Editor:*

A recent investigation by Cheong and colleagues should pique the interest of all clinicians who employ sonography during resuscitation [1]. In their report, a novel method of measuring the left common carotid artery, maximum velocity time integral (VTI_MAX-CA_) was described and its value was related to the left ventricular outflow tract VTI (VTI_LVOT_). Absolute VTI measurements (in centimeters) were made in critically-ill patients, though the population studied was relatively stable, seemingly not on vasoactive medications and with normal cardiac function. Importantly, there was no provocative (i.e., dynamic) maneuver carried out during their investigation.

As anticipated, Cheong and colleagues observed a stronger relationship between total (i.e., systolic plus diastolic) VTI_MAX-CA_ and VTI_LVOT_ than between only the systolic portion of the VTI_MAX-CA_ and the VTI_LVOT_. Of most interest, however, was the near parity between VTI_MAX-CA_ and VTI_LVOT_ in absolute value. Based on their regression equation, the VTI_MAX-CA_ overestimated the VTI_LVOT_ less than 10%. Considering why this might be so elaborates some caveats to their approach.

## The maximum-to-centroid velocity ratio

What escapes some clinical sonographers is that the VTI of hemodynamic interest is not the maximum VTI, but rather the ‘centroid’ VTI (VTI_CENT_). The centroid velocity is a ‘power weighted,’ *average velocity* across the vessel lumen [2–4]. Importantly, the relationship between VTI_CENT_ and the maximum VTI (VTI_MAX_) depends upon the velocity profile within the vessel [2, 3]. In ‘plug flow’ conditions (e.g., LVOT, ascending aorta), the velocity profile is flat such that maximum and centroid velocities are nearly identical [5]. Accordingly, the maximum-to-centroid ratio is roughly 1.0 at the LVOT. By contrast, ‘parabolic flow’ is characterized by a maximum velocity double that of the centroid velocity (i.e., a max-to-centroid ratio of 2.0) [2]. This occurs in smaller-diameter vessels where the centerline red blood cell (RBC) velocity is greatest and there is progressive slowing of the RBCs towards the lumen periphery; however, few vessels in the body are characterized by fully-developed, parabolic flow [5]. The velocity profile of the carotid artery, for instance, is characterized as ‘blunted parabolic,’ with a max-to-centroid ratio approximately mid-way between 1.0 and 2.0 [4]. Given the above, we can express the following relationship as Eq. (1).

1 $$ K = \frac{{VTI_{MAX} }}{{VTI_{CENT} }} $$

where *K* = 1.0 in plug flow; *K* = 2.0 in parabolic flow and *K* $\approx$ 1.5 in blunted parabolic flow.

Using the wireless, wearable Doppler system developed by our group [6–10], we have observed that in resting, healthy volunteers, the common carotid artery max-to-centroid ratio falls between 1.5 and 1.7 over the entire cardiac cycle. Thus, for simplicity we assume that the VTI_MAX-CA_ is 1.6 times the carotid artery centroid VTI (VTI_CENT-CA_); that is, *K* = 1.6 and we express Eq. (2):

2 $$ VTI_{MAX - CA} = 1.6 \; \times \;VTI_{CENT - CA} . $$

Furthermore, we assume that the velocity profile in the left ventricular outflow tract is plug; thus, Eq. 3:

3 $$ VTI_{MAX - LVOT} = 1.0 \times VTI_{CENT - LVOT} . $$

In other words, the LVOT maximal velocity is used interchangeably with the LVOT centroid velocity.

## Relationship between LVOT and carotid artery VTI

The stroke volume (in mL or cm^3^) is calculated with ultrasound by multiplying the cross-sectional area (CSA) of the LVOT (in cm^2^) by the VTI_LVOT_ (in cm) (Eq. 4) [11]:

4 $$ SV = CSA_{LVOT} \; \times \;VTI_{LVOT} . $$

The volume of the SV that moves up a carotid artery, the carotid beat volume (CBV), can be generally expressed as the fraction of the SV distributed to one carotid artery (CA_FLOWFRAC_). The CBV can also be calculated analogously to the SV, by multiplying the CSA of the carotid artery (CSA_CA_) by the VTI_CENT-CA_. Therefore, we arrive at Eq. (5):

5 $$ CBV = CSA_{CA} \times VTI_{CENT - CA} = CA_{FLOWFRAC} \times SV. $$

By substituting Eq. (4) (for SV) into Eq. (5) above, and rearranging, we arrive at Eq. (6):

6 $$ VTI_{CENT - CA} = \frac{{CSA_{LVOT} }}{{CSA_{CA} }} \times CA_{FLOWFRAC} \times VTI_{LVOT} $$

And finally, to convert VTI_CENT-CA_ to VTI_MAX-CA_, which was the measurement obtained by Cheong and colleagues, we derive Eq. (7):

7 $$ VTI_{MAX - CA} = K \times \left[ { \frac{{CSA_{LVOT} }}{{CSA_{CA} }} \times CA_{FLOWFRAC} \times VTI_{LVOT} } \right] $$

where *K* = 1.6

## Clinical implications

To make this more concrete, we might consider plugging in some typical anthropometric values into Eq. (7). For example, if typical CSA_LVOT_ [12] and CSA_CA_ [13] values are 3.6 cm^2^ and 0.36 cm^2^, respectively, then the CSA_LVOT_-to-CSA_CA_ ratio is roughly 10. Curiously, a reasonable approximation of the CA_FLOWFRAC_ is 0.10 [14], meaning that the CSA_LVOT_-to-CSA_CA_ ratio and CA_FLOWFRAC_ reduce to 1.0. Nevertheless, as detailed above, the maximum velocity in the carotid artery is greater than its centroid; thus, we expect the VTI_MAX-CA_ to be greater than the VTI_LVOT_ as a function of the velocity profile (i.e., *K* = 1.6). One speculative explanation for the very slight overestimation observed by Cheong and colleagues is their novel method of insonating the left carotid artery. They ‘looked down’ from the supraclavicular fossa and may have insonated near the bifurcation of the left common carotid artery from the aortic arch. Velocity profiles at sharp bifurcations behave in complicated ways [2], but the profile can be flat near the origin, especially if the mother vessel is large like the aorta. The profile in the smaller vessel then evolves a parabolic morphology only after a distance known as the ‘entrance length,’ which is estimated as roughly 10 cm for the carotid arteries [2]. Thus, insonating near the origin of the left carotid artery may have reduced *K* towards a ‘plug’ profile value (i.e., *K* = 1.1 or 1.2) which would make the VTI_MAX-CA_ closer in absolute value to the VTI_LVOT._

Regardless of the above, the clinical implications of Eq. (7) are probably greater for something Cheong et al. did not do, that is, perform a hemodynamic intervention. When doing so, the clinician is typically trying to infer change in the VTI_LVOT_ via the VTI_MAX-CA_ We see, however, that two variables in particular (i.e., the CSA_CA_, and CA_FLOWFRAC_) may co-vary during an intervention and thus dissociate the VTI_MAX-CA_ from the VTI_LVOT_.

First, with provision of intravenous fluid, the CSA_CA_ can increase [15]. This may be especially important in hypotensive patients in whom increased in mean arterial pressure affects relatively large vessel distension [16]. Per Eq. (7), augmented CSA_CA_ causes the VTI_MAX-CA_ to *underestimate* the VTI_LVOT_.

Second, an intervention that also changes the CA_FLOWFRAC_ would also cause VTI_MAX-CA_ to diverge from the VTI_LVOT_. Fundamentally, the CA_FLOWFRAC_ is directly proportional to the ratio of whole-body-to-head vascular impedance [6]. For example, lowering body-to-head impedance diminishes CA_FLOWFRAC_. An illustration of this is exercise, where muscles vasodilate and ‘siphon’ blood away from the head. This was shown in the study of Sato and colleagues where baseline CA_FLOWFRAC_ was about 0.14 and fell to about 0.06 at peak exercise [17]. Ostensibly, inodilators have a similar effect; per Eq. (7), when CA_FLOWFRAC_ falls, VTI_MAX-CA_ *underestimates* VTI_LVOT_. On the other hand, increased body-to-head vascular impedance raises CA_FLOWFRAC_ and causes the VTI_MAX-CA_ to *overestimate* VTI_LVOT_. Catecholamines, which preferentially vasoconstrict ‘non-essential’ blood flow to maintain brain and coronary perfusion have this effect. This was recently observed by Kim and colleagues where carotid blood flow increased relative to cardiac output in response to norepinephrine [18]. Though catecholamines are the most commonly employed intervention that raises body-to-head impedance, mechanical therapies such as resuscitative endovascular balloon occlusion of the aorta (i.e., REBOA) and intra-aortic counter-pulsation would have similar hemodynamic effects.

Finally, within Eq. (7) we can reasonably assume constancy of the CSA_LVOT_ during most interventions, though the value of *K*, in theory, might decrease with CSA_CA_. This is because the Womersley equation predicts flatter velocity profiles (i.e., decreasing *K*) with increasing vessel diameter [19]. Thus, carotid artery vessel distention has multiple mechanisms by which VTI_MAX-CA_ *underestimates* VTI_LVOT_.

In summary, Cheong and colleagues are to be congratulated for their impressive clinical work and their novel approach to carotid insonation. As shown in Eq. 7, there is a direct relationship between VTI_LVOT_ and VTI_MAX-CA_. However, vessel distension, CA_FLOWFRAC_ and velocity profile will mediate this link and these covariates may be especially important during hemodynamic interventions where the clinician performs pre-post VTI calculations. Furthermore, the framework discussed above could be applied to peripheral arteries other than the carotid. Novel means to infer real-time vessel diameter, body-to-head impedance and velocity profile will better model the association between the left ventricle and common carotid artery, especially in conjunction with other Doppler measures such as the corrected flow time [6].

## Data Availability

Not applicable.
